# Dimensionality and Dynamics in the Behavior of *C. elegans*

**DOI:** 10.1371/journal.pcbi.1000028

**Published:** 2008-04-25

**Authors:** Greg J. Stephens, Bethany Johnson-Kerner, William Bialek, William S. Ryu

**Affiliations:** 1 Lewis–Sigler Institute for Integrative Genomics, Princeton, New Jersey, United States of America; 2 Joseph Henry Laboratories of Physics, Princeton, New Jersey, United States of America; 3 Center for the Study of Brain, Mind and Behavior, Princeton University, Princeton, New Jersey, United States of America; Indiana University, United States of America

## Abstract

A major challenge in analyzing animal behavior is to discover some underlying simplicity in complex motor actions. Here, we show that the space of shapes adopted by the nematode *Caenorhabditis elegans* is low dimensional, with just four dimensions accounting for 95% of the shape variance. These dimensions provide a quantitative description of worm behavior, and we partially reconstruct “equations of motion” for the dynamics in this space. These dynamics have multiple attractors, and we find that the worm visits these in a rapid and almost completely deterministic response to weak thermal stimuli. Stimulus-dependent correlations among the different modes suggest that one can generate more reliable behaviors by synchronizing stimuli to the state of the worm in shape space. We confirm this prediction, effectively “steering” the worm in real time.

## Author Summary

A great deal of work has been done in characterizing the genes, proteins, neurons, and circuits that are involved in the biology of behavior, but the techniques used to quantify behavior have lagged behind the advancements made in these areas. Here, we address this imbalance in a domain rich enough to allow complex, natural behavior yet simple enough so that movements can be explored exhaustively: the motions of *Caenorhabditis elegans* freely crawling on an agar plate. From measurements of the worm's curvature, we show that the space of natural worm postures is low dimensional and can be almost completely described by their projections along four principal “eigenworms.” The dynamics along these eigenworms offer both a quantitative characterization of classical worm movement such as forward crawling, reversals, and Omega-turns, and evidence of more subtle behaviors such as pause states at particular postures. We can partially construct equations of motion for this shape space, and within these dynamics we find a set of attractors that can be used as a rigorous definition of behavioral state. Our observations of *C. elegans* reveal a precise and complete language of motion and new aspects of worm behavior. We believe this is a lesson with promise for other organisms.

## Introduction

The study of animal behavior is rooted in two divergent traditions. One approach creates well-controlled situations, in which animals are forced to choose among a small discrete set of behaviors, as in psychophysical experiments [1]. The other, taken by ethologists [2], describes the richness of the behaviors seen in more natural contexts. One might hope that simpler organisms provide model systems in which the tension between these approaches can be resolved, leading to a fully quantitative description of complex, naturalistic behavior.

Here we explore the motor behavior of the nematode, *Caenorhabditis elegans*, moving freely on an agar plate [3]–[9]. Though lacking the full richness of a natural environment, this unconstrained motion allows for complex patterns of spontaneous motor behaviors [10], which are modulated in response to chemical, thermal and mechanical stimuli [11]–[13]. Using video microscopy of the worm's movements, we find a low dimensional but essentially complete description of the macroscopic motor behavior. Within this low dimensional space we reconstruct equations of motion which reveal multiple attractors—candidates for a rigorous definition of behavioral states. We show that these states are visited as part of a surprisingly reproducible response of *C. elegans* to small temperature changes. Correlations among fluctuations along the different behavioral dimensions suggest that some of the randomness in the behavioral responses could be removed if sensory stimuli are delivered only when the worm is at a well defined initial state. We present experimental evidence in favor of this idea, showing that worms can be “steered” in real time by appropriately synchronized stimuli.

## Results

### Eigenworms

**Figure 1:**
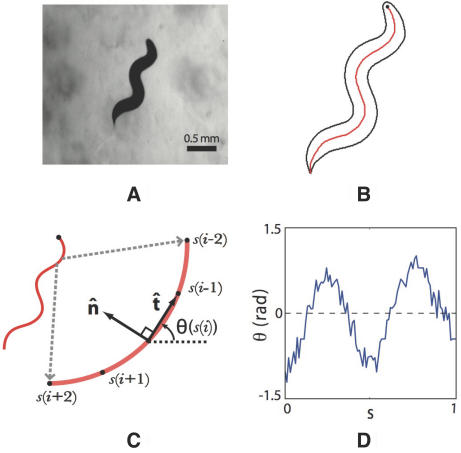
Describing the shapes of worms. (A) Raw image in the tracking microscope. (B) The curve through the center of the body. The black circle marks the head. (C) Distances along the curve (arclength *s*) are measured in normalized units, and we define the tangent 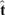
(*s*) and normal 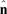
(*s*) to the curve at each point. The tangent points in a direction *θ*(*s*), and variations in this angle correspond to the curvature κ(*s*) = *d*θ(*s*)/*ds*. (D) All images are rotated so that 〈*θ*〉 = 0; therefore *θ* (*s*) provides a description of the worm's shape that is independent of our coordinate system, and intrinsic to the worm itself.

We use tracking microscopy with high spatial and temporal resolution to extract the two-dimensional shape of individual *C. elegans* from images of freely moving worms over long periods of time (Figure 1A; see Materials and Methods). Variations in the thickness of the worm are small, so we describe the shape by a curve that passes through the center of the body (Figure 1B). We measure position along this curve (arc length) by the variable *s*, normalized so that *s* = 0 is the head and *s* = 1 is the tail. The position of the body element at *s* is denoted by **x**(*s*), and we sample this function at *N* = 100 equally spaced points along the body. These variables provide an essentially complete description of the motor output.

We analyze the worm's shapes in a way intrinsic to its own behavior, not to our arbitrary choice of coordinates (Figure 1). The intrinsic geometry of a curve in the plane is defined by the Frenet equations [14],[15],
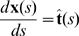
(1)

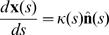
(2)where 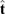
(*s*) is the unit tangent vector to the curve, 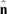
(*s*) is the unit normal to the curve, and κ(*s*) is the scalar curvature. If the tangent vector points in a direction κ(*s*), then κ(*s*) = *d*θ(*s*)/*ds*. Curvature as a function of arc length, *θ*(*s*), thus provides a “worm–centered” description, but in practice this involves taking two derivatives and thus is noisy. As an alternative, we describe the curve by *θ*(*s*), but remove the dependence on our choice of coordinates by rotating each image so that the mean value of *θ* along the body always is zero; this rotated version of *θ*(*s*) contains exactly the same information as κ(*s*).

**Figure 2:**
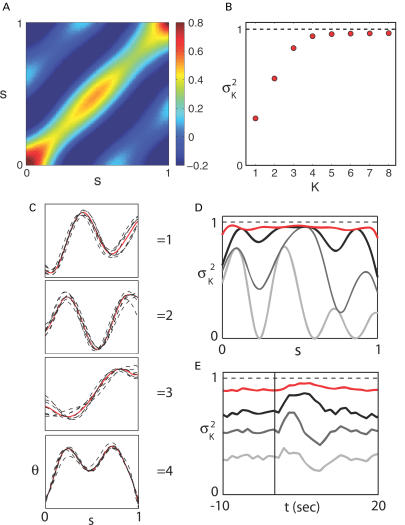
Covariance of shape fluctuations and eigenworms. (A) The covariance matrix of fluctuations in angle *C*(*s*, *s*′). The inhomogeneity along the diagonal shows that the normal modes of the motion are not sinusoidal but the smooth structure of *C*(*s*, *s*′) means that a small number of modes are significant. (B) We find the eigenvalues of *C*(*s*, *s*′) and compute *σ* ^2^ *_K_*, the fraction of the total variance (integrated along the body of the worm) captured by keeping *K* modes (see Materials and Methods). (C) Associated with each dominant mode is an eigenvector and we refer to these as eigenworms *u*_μ_(s). The population-mean eigenworms (red) are highly reproducible across individual worms (black). (D) The fraction of variance, *σ̃* ^2^ *_K_*, at each point along the body curve captured by keeping *K* modes (*K* = 1 to 4, from bottom to top curve). The overall error in reconstruction of the worm body curve decreases as the number of modes increases, but does so inhomogeneously. (E) In response to strong thermal stimuli, reconstructions using the eigenworms of spontaneous crawling continue to account for most of the shape variance. Worm images are recorded at times synchronized to a heat pulse and we display *σ* ^2^ *_K_* aligned with this pulse (red line). (*K* = 1 to 4, from bottom to top curve).

Although the worm has no discrete joints, we expect that the combination of elasticity in the worm's body wall and a limited number of muscles will lead to a limited effective dimensionality of the shape and motion. In the simplest case, the relevant low dimensional space will be a Euclidean projection of the original high dimensional space. If this is true, then the covariance matrix of angles, *C*(*s*, *s*′) = 〈(*θ*(*s*)–〈*θ*〉)(*θ*(*s*′)–〈*θ*〉)〉 will have only a small number of nonzero eigenvalues. Figure 2A shows the covariance matrix, and its smooth structure is a strong hint that there will be only a small number of significant eigenvalues; this is shown explicitly in Figure 2B. Quantitatively, over 95% of the total variance in angle along the body is accounted for by just four eigenvalues. Note that the contribution of the variance is inhomogeneous along the body curve. For example the fourth eigenworm makes a small contribution to the variance overall, but captures a large percentage of the variance within 5% of the head and tail region (Figure 2D).

Associated with each of the eigenvalues *λ*_μ_ is an eigenvector *u*_*μ*_(*s*), sometimes referred to as a ‘principal component’ of the function *θ*(*s*). If only *K* = 4 eigenvalues are significant, then we can write the shape of the worm as a superposition of ‘eigenworm’ shapes,
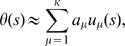
(3)where the four variables {*a*_μ_} are the amplitudes of motion along the different principal components, 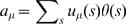. We see in Figure 2C that these modes are highly reproducible from individual to individual.

Thus far we have considered only worms moving in the absence of deliberate sensory stimuli. Do the worms continue to move in just a four dimensional shape space when they respond to strong inputs? To test this, we delivered intense pulses of heat (see Materials and Methods), which are known to trigger escape responses [16]. We see in Figure 2D that we still account for ≈95% of the shape variance using just four modes, even though the distribution of shapes during the thermal response is very different from that seen in spontaneous crawling. We conclude that our four eigenworms provide an effective, low dimensional coordinate system within which to describe *C. elegans* motor behavior.

### What Do the Modes Mean?

**Figure 3:**
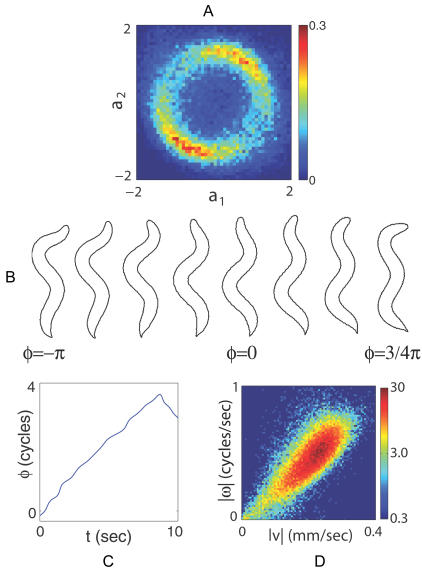
Motions along the first two eigenworms. (A) The joint probability density of the first two amplitudes, ρ(*a*_1_, *a*_2_), with units such that 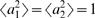. The ring structure suggests that these modes form an oscillator with approximately fixed amplitude and varying phase *φ* = tan^-1^(-*a*_2_/*a*_1_). (B) Images of worms with different values of *φ* show that variation in phase corresponds to propagating a wave of bending along the worm’s body. (C) Dynamics of the phase *φ*(*t*) shows long periods of linear growth, corresponding to a steady rotation in the {*a*_1_, *a*_2_} plane, with occasional, abrupt reversals. (D) The joint density ρ(|*ν*|,|*ω*|). The phase velocity *ω* = *dφ*/*dt* in shape space predicts worm’s crawling speed.

The projection of worm shapes onto the low-dimensional space of eigenworms provides a new and quantitative foundation for the classical, qualitative descriptions of *C. elegans* behavior [10]. The first two modes are sinuous (although not exactly sinusoidal) oscillations of the body shape (Figure 2C); they form a quadrature pair, so that different mixtures of the two modes correspond to different phases of a wave along the body. Indeed, the probability distribution of the mode amplitudes, *ρ*(*a*_1_, *a*_2_), shows a ring of nearly constant amplitude (Figure 3A). Sampling images around this ring reveals a traveling wave along the body (Figure 3B). There are relatively long periods of time where the shape changes by a continuous accumulation of the phase angle (Figure 3C), and the speed of this rotation predicts the speed at which the worm crawls (Figure 3D).

**Figure 4:**
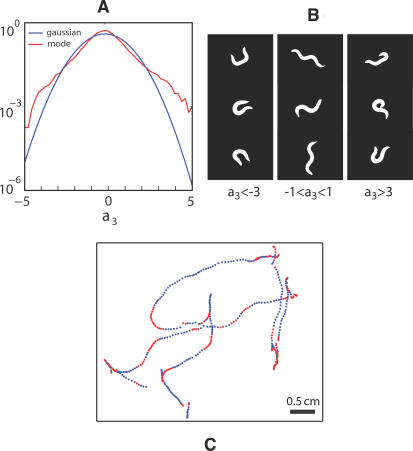
Motions along the third eigenworm. (A) The distribution of amplitudes *ρ*(*a*_3_), shown on a logarithmic scale. Units are such that 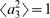, and for comparison we show the Gaussian distribution; note the longer tails in *ρ*(*a*_3_). (B) Images of worms with values of *a*_3_ in the negative tail (left), the middle (center) and positive tail (right). Large negative and positive amplitudes of *a*_3_ correspond to bends in the dorsal and ventral direction, respectively. (C) A two minute trajectory of the center of mass sampled at 4 Hz. Periods where |*a*_3_|>1 are colored red, illustrating the association between turning and large displacements along this mode.

In contrast to the first two modes, the third mode *u*_3_(*s*) contributes to a nearly constant curvature throughout the middle half of the body (Figure 2C). The distribution of the mode amplitude *a*_3_ has a long tail (Figure 4A), and body shapes chosen from these tails (Figure 4B) exhibit the Ω configuration classically identified with large-angle turning behavior [10]. Large amplitudes of *a*_3_ also correspond to gradual turns in the worm trajectory along the agar (Figure 4C).

The fourth mode *u*_4_(*s*) contributes to the shape of the head and tail region of the worm. Figure 2D shows that *u*_4_(*s*) captures a large amount of the shape variance in those regions. Head movements of the worm are likely important in foraging [17] and navigation [18]. The emergence of a separate mode is likely due to the fact that head of *C. elegans* can move independently of the body and is controlled by a separate set of neck muscles.

The connections between mode amplitudes and the motion of the worm along the agar—as in Figures 3D and 4C—are genuine tests of the functional meaning of our low dimensional description. Quite explicitly, our analysis of worm shapes is independent of the extrinsic coordinates and hence our definition of modes and amplitudes is blind to the actual position and orientation of the worm. Of course, in order to move the worm must change shape, and our description of the shape in terms of mode amplitudes captures this connection to movement. Thus, to crawl smoothly forward or backward the worm changes its shape by rotating clockwise or counterclockwise in the plane formed by the mode amplitudes *a*_1_ and *a*_2_; the speed of crawling is set by the speed of the rotation. Similarly, to change direction the worm changes shape toward larger magnitudes of the mode amplitude *a*_3_, and we see this connection even without defining discrete turning events.

### Attractors and Behavioral States

The eigenworms provide a coordinate system for the postures adopted by *C. elegans* as it moves; to describe the dynamics of movement we need to find equations of motion in this low dimensional space. We start by focusing on the plane formed by the first two mode amplitudes *a*_1_ and *a*_2_. Figure 3 suggests that within this plane the system stays at nearly constant values of the radius, so that the relevant dynamics involves just the phase angle *φ*(*t*). To account for unobserved and random influences these equations need to be stochastic, and to support both forward and backward motion they need to form a system of at least second order. Such a system of equations would be analogous to the description of Brownian motion using the Langevin equation [19],[20]. Thus we search for equations of the form
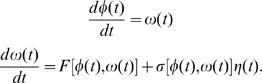
(4)


Here *F*[*φ*(*t*),*ω*(*t*)] defines the average acceleration as a function of the phase and phase velocity, by analogy to the force on a Brownian particle. The noise is characterized by a random function *η*(*t*) which we hope will have a short correlation time, and we allow the strength of the noise *F*[*φ*(*t*),*ω*(*t*)] to depend on the state of the system, by analogy to a temperature that depends on the position of the Brownian particle.

**Figure 5:**
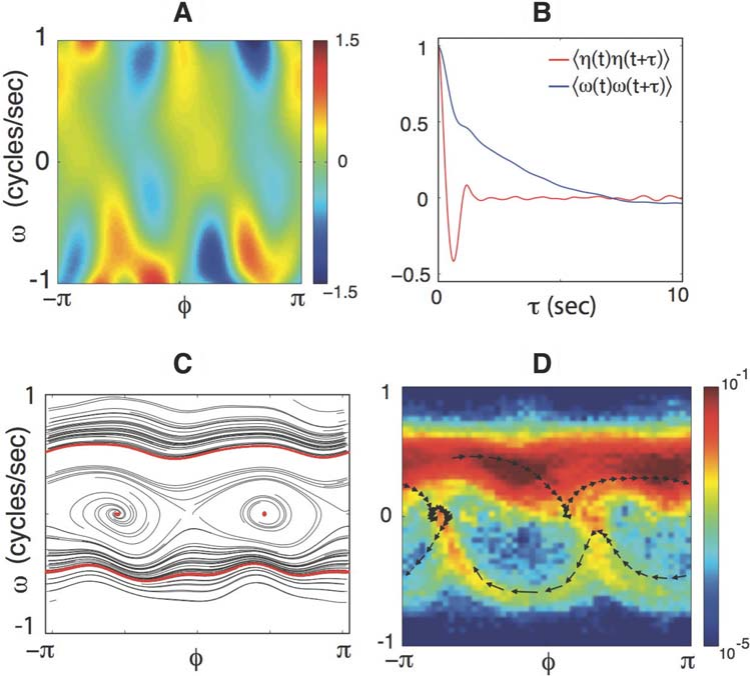
Reconstructing the phase dynamics. (A) The mean acceleration of the phase *F*(*ω,φ*) in Equation 4. (B) The correlation function of the noise 〈*η* (*t*)*η*(*t*+*τ*)〉. The noise correlations are confined to short times relative to the phase velocity itself. (C) Trajectories in the deterministic dynamics. A selection of early-time trajectories is shown in black. At late times these same trajectories collapse to one of four attractors (red): forward and backward crawling and two pause states. (D) Joint density *ρ*(*ω*,*φ*) for worms sampled at 32 Hz. A sample trajectory of a single worm moving forwards, backwards, and pausing, is denoted by black arrows.

In Figure 5A we show our best estimate of the mean acceleration *F*[*φ*,*ω*] (see Materials and Methods for details). Once we know *F*, we subtract this mean acceleration from the instantaneous acceleration to recover trajectories of the noise, and the correlation function of this noise is shown in Figure 5B. The correlation time of the noise is short, which means that we have successfully separated the dynamics into two parts: a deterministic part, described by the function *F*[*φ*,*ω*], which captures the average motion in the {*a*_1_,*a*_2_} plane and hence the relatively long periods of constant oscillation, and a rapidly fluctuating part *η*(*t*) that describes “jittering” around this simple oscillation as well as the random forces that lead to jumps from one type of motion to another.

We can imagine a hypothetical worm which has the same deterministic dynamics as we have found for real worms, but no noise. We can start such a noiseless worm at any combination of phase and phase velocity, and follow the dynamics predicted by Equation 4, but with *σ* = 0. These dynamics are diverse on short time scales, depending in detail on the initial conditions, but eventually all initial conditions lead to one of a small number of possibilities (Figure 5C): either the phase velocity is always positive, always negative, or decays to zero as the system pauses at one of two stationary phases. Thus, underneath the continuous, stochastic dynamics we find four discrete attractors which correspond to well defined classes of behavior.

We can compare the predicted behavioral states with the motion of real worms that include transitions between these states. Figure 5D is the joint probability density, *ρ*(*ω,φ*), of worms sampled at 32 Hz; the trajectory of a single worm visiting all three predicted behavioral states is indicated by the overlay. The forward (*ω*>0) and backward (*ω*<0) motions match well with previously calculated attractor states, and pauses in the trajectory of real worms correspond to the calculated pause basins (*ω* = 0). Surprisingly, the transition between forward and backward motion is not arbitrary, but occurs most often along specific phase dependent trajectories.

### Pause States and Reproducibility

The behavior of *C. elegans*, particularly in response to sensory stimuli, traditionally has been characterized in probabilistic terms: worms respond by changing the probability of turning or reversing [17],[21],[22]. This randomness could reflect an active strategy on the part of the organism, or it could reflect the inability of the nervous system to distinguish reliably between genuine sensory inputs and the inevitable background of noise. Our description of motor behavior measured with high time resolution offers us the opportunity to revisit the “psychophysics” of *C. elegans*.

**Figure 6:**
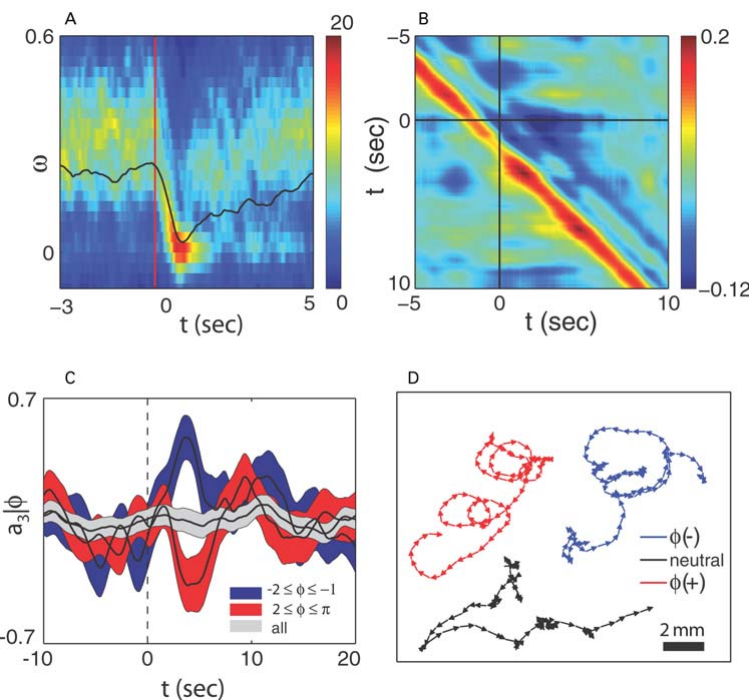
Thermal responses, mode coupling and active steering. (A) The distribution of phase velocities *ρ_t_*(*ω*) in response to a brief thermal stimulus. Within one second, the distribution becomes highly concentrated near *ω = *0, corresponding to the pause states identified in Figure 5. (B) Correlations between phase in the {*a*_1_,*a*_2_} plane and *a*_3_, 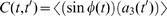. Shortly after the thermal impulse (*t*, *t*′>0) the modes develop a strong anti-correlation which is distinct from normal crawling. (C) Phase dependent thermal response. Worms stimulated during ventral head swings (−2≤*φ*≤−1) turn dorsally (red) while worms stimulated during dorsal head swings (2≤*φ*≤π) turn ventrally (blue). When phase is ignored there is no discernible response (grey). Solid lines denote averages while colored bands display standard deviation of the mean. (D) Worm “steering.” A thermal impulse conditioned on the instantaneous phase was delivered automatically and repeatedly, causing an orientation change 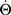 in the worm's trajectory. In this example lasting 4 minutes, asynchronous impulses produced a time-averaged orientation change 〈
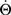
〉 = 0.01 rad/s (black), impulses at positive phase produced a trajectory with 〈
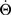
〉 = 0.10 rad/s (blue), and impulses at negative phase produced 〈
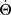
〉 = –0.12 rad/s (red). This trajectory response is consistent with the mode correlations seen in Figure 6C. We found 13 out of 20 worms produced statistically different orientation changes under stimulated and non-simulated conditions while only 1 out of 20 worms responded in the same fashion when the phase was randomized (*p*<0.01, Fisher exact test).

We consider the response to brief (75 ms), small (Δ*T*≈0.1°C) changes in temperature, induced by pulses from an infrared laser (see Materials and Methods). These stimuli are large enough to elicit responses [12] but well below the threshold for pain avoidance [16]. In Figure 6 we show the distribution *ρ*_t_(*ω*) of phase velocities as a function of time relative to the thermal pulse. All of the worms were crawling forward at the moment of stimulation, so the initial phase velocities are distributed over a wide range of positive values. Within one second, the distribution narrows dramatically, concentrating near zero phase velocity. This behavior is consistent with the worm visiting the pause states described above in the deterministic dynamics, and may be similar to the pausing response seen when worms are subjected to mechanical stimuli [23].

Arrival in the pause state is stereotyped both across trials and across worms. By analogy with conventional psychophysical methods [1], we can ask how reliably an observer could infer the presence of the heat pulse using the worm's response. We find that just measuring the phase velocity *ω* at single moment in time after the pulse is sufficient to provide ≈75% correct detection of this small temperature change in single trials.

### Coupling the Modes and Steering the Worm

Our discussion thus far has separated the dynamics of the worm into two very different components: the {*a*_1_, *a*_2_} plane with its phase dynamics, responsible for crawling motions, and the mode *a*_3_, which is connected with large curvature turns. Because these modes are eigenvectors of a covariance matrix their instantaneous amplitudes are not linearly correlated, but this does not mean that the dynamics of the different motions are completely uncoupled. We found the clearest indications of mode coupling between the phase in the {*a*_1_, *a*_2_} plane and the amplitude *a*_3_ at later times, which is illustrated by the correlation function in Figure 6B. The diagonal band of positive correlation reflects the phase dependent bending motions of normal crawling. This pattern of correlations is perturbed strongly by thermal stimuli (*t*, *t*′>0). The fact that the correlations between phase and the turning mode are stimulus dependent implies that the response of the turning mode to thermal stimuli depends on the phase which the worm finds itself at the time of the stimulus. Perhaps some of the apparent randomness of turning responses thus is related to the fact that repeated thermal stimuli catch the worm at different initial phases. To test this idea, Figure 6C shows the average response of *a*_3_ when worms are thermally stimulated with their head turned to either the dorsal or ventral side. Worms stimulated when making a ventral head swing (−2≤*φ*≤−1) make bends in the dorsal direction (*a*_3_<0), and vice versa. Note that the thermal pulse itself does not have a handedness, so that if the pulses are not synchronized to the state of the worm there should be no systematic preference for dorsal vs. ventral handed turns. As a further test of this idea, we implemented our analysis online, allowing an estimate of the phase with a delay of less than 125 ms. We then deliver an infrared pulse when the phase falls within a phase window that corresponds to either dorsal– or ventral– directed head swings. The predicted consequence is that the worm should turn in the opposite direction to the laser stimulation, and is confirmed in Figure 6D.

## Discussion

Our central result is a new, quantitative, and low-dimensional description of *C. elegans* motor behavior. Conceptually similar results have been obtained for aspects of motor control in humans and other primates, where postures or trajectories of limbs, hands or eyes are confined to spaces of low dimensionality despite the potential for more complex motions [24]–[27]. For *C. elegans* itself, recent quantitative work has focused on simplifying behavior by matching to a discrete set of template behaviors, such as forward and backward motion of the center of mass [5], sinusoidal undulations of the body [6], or Ω bends [7]. Our results combine and generalize these ideas. Motor behaviors are described by projection of the body shape onto a small set of templates (the eigenworms), but the strengths of these projections vary continuously. The templates are sinuous, but not sinusoidal, because the fluctuations in posture are not homogeneous along the length of the worm. Our description of shape is intrinsic to the worm and invariant to the center of mass position, but motion in shape space predicts the center of mass motion. There are discrete behavioral states, but these emerge as attractors of the underlying dynamics. Most importantly, our choice of four eigenworms is driven not by hypotheses about the relevant components of behavior, but by the data itself.

The construction of the eigenworms guarantees that the instantaneous amplitudes along the different dimensions of shape space are not correlated linearly, but the dynamics of the different amplitudes are nonlinear and coupled; what we think of as a single motor action always involves coordinating multiple degrees of freedom. Thus, forward and backward motion correspond to positive and negative phase velocity in Figure 3, but transitions between these behavioral states occur preferentially at particular phases. Similarly, turns involve large amplitude excursions along *a*_3_, but motion along this mode is correlated with phase in the ({*a*_1_, *a*_2_}) plane, and this correlation itself has structure in time (Figure 6B). The problems of *C. elegans* motor control are simpler than for higher animals, but these nonlinear, coupled dynamics give a glimpse of the more general case.

Perhaps because of the strong coupling between the turning mode *a*_3_ and the wriggling modes *a*_1_, *a*_2_, we have not found an equation of motion for *a*_3_ alone which would be analogous to Equation 4 for the phase. Further work is required to construct a fully three dimensional dynamics which could predict the more complex correlations such as those in Figure 6B. Turning should emerge from these equations not as another attractor, but as an ‘excitable’ orbit analogous to the action potential in the Hodgkin–Huxley equations or to recent ideas about transient differentiation in genetic circuits [28]. A major challenge would be to show that the stochastic dynamics of these equations can generate longer sequences of stereotyped events, such as pirouettes [29].

We have shown that a meaningful set of behavioral coordinates can uncover deterministic responses. A response might seem stochastic or noisy because it depends on one or more behavioral variables that are not being considered. In our experiments, nonlinear correlations among the behavioral variables suggest that some of the randomness in behavioral responses could be removed if sensory stimuli are delivered only when the worm is at a well defined initial state, and we confirmed this prediction by showing that phase–aligned thermal stimuli can ‘steer’ the worm into trajectories with a definite chirality. A crucial aspect of these experiments is that the stimulus is scalar—a temperature change in time has no spatial direction or handedness—but the response, by virtue of the correlation between stimulus and body shape, does have a definite spatial structure. The alignment of thermal stimuli with the phase of the worm's movement in these experiments mimics the correlation between body shape and sensory input that occurs as the worm crawls in a thermal gradient, so the enhanced determinism of responses under these conditions may be connected to the computations which generate nearly deterministic isothermal tracking [22],[30].

More generally, all behavioral responses have some mixture of deterministic and stochastic components. In humans and other primates, it seems straightforward to create conditions that result in highly reproducible, stereotyped behaviors, such as reaching movements [31]. At the opposite extreme, bacterial motility is modulated in response to sensory inputs, but these responses seem fundamentally probabilistic [32]. Some of these differences may result from the physical nature of sensory stimuli in organisms of vastly different size [33],[34], but some of the differences may also result from differences of strategy or available computational power. The more stochastic the response, the more challenging it is to characterize behavior quantitatively and to link behavior with underlying molecular and neural components, as is clear from recent work on *Drosophila* olfaction (see, for example, [35]). We hope that our approach to the analysis of behavior may help to uncover more deterministic components of the sensory–motor responses in other model organisms.

More than forty years of work on *C. elegans* has led to a fully sequenced genome [36] and to the complete wiring diagram of the nervous system [37]. Significant steps have been made toward the original dream [38] of connecting genes, neurons, and behavior [11],[39],[40]. Nonetheless, with the advances in molecular, cellular, and circuit analyses, our ability to probe the mechanisms which generate behavior substantially exceeds our ability to characterize the behavior itself. Perhaps our work provides a step toward addressing this imbalance.

## Materials and Methods

### Tracking Microscopy

The imaging system consists of a Basler firewire CMOS camera (A601f, Basler, Ahrensburg, Germany) with 4x lens (55–901, Edmund Optics, Barrington, NJ) and a fiber optic trans-illuminator (DC-950, Dolan-Jenner, Boxborough, MA) mounted to an optical rail (Thorlabs, Newton, NJ). The rail is attached to a XY translation stage (Deltron, Bethel, CT) which is driven by stepper motors (US Digital, Vancouver, Washington). The stage driver is a homemade unit utilizing a SimpleStep board (SimpleStep, Newton, NJ) and Gecko stepper motor drivers (Geckodrive, Santa Ana, CA). Image acquisition, processing, and stage driver control was done using LabVIEW (National Instruments, Austin, TX). Images of worms were isolated and identified using the image particle filter. A raw unprocessed JPEG image and a filtered process binary PNG image were written to the hard drive at rates up to 32 Hz. Concurrently at 4 Hz, the center of mass of the worm was calculated and the distance from the center of the field of view in pixels was computed. An error signal was then calculated via a coordinate transformation between the camera reference frame and the translational stage reference frame and the XY stage was moved to center the worm in the field of view.

### Worm Preparation

The *C. elegans* strain, N2, was grown at 20°C and maintained under standard conditions [41]. Before each experiment, excess moisture from NGM assay plates (1.7% Bacto Agar, 0.25% Bacto-Peptone, 0.3% NaCl, 1 mM CaCl_2_, 1 mM MgSO_4_, 25 mM potassium phosphate buffer, 5 µg/mL cholesterol) was removed by leaving them partially uncovered for 1 hr. A copper ring (5.1-cm inner diameter) pressed into the agar surface prevented worms from crawling to the side of the plate. Young adults were rinsed of *E. coli* by transferring them with a worm pick from OP50 bacterial food plates into NGM buffer (same inorganic ion concentration as NGM assay plates) and letting them swim for 1 minute. Worms were transferred from the NGM buffer to the center of the assay plate (9-cm Petri dish). The location of the dorsal side of the worm was noted via a stereomicroscope. The plates were covered and tracking began after 1 minute and lasted no longer than 60 minutes. In the rare cases where worms stopped moving before the completion of the run, the data were excluded.

### Eigenworms

Images of worms captured by the worm tracker were processed using MATLAB (Mathworks, Natick, MA). Cases of self-intersection were excluded from processing. Images of worms were thinned to a single-pixel-thick backbone, and aligned so that the dorsal/ventral directions were consistent. A spline was fit through these points and then discretized into 101 segments, evenly spaced in units of the backbone arclength. The *N* = 100 angles between these segments were calculated and an overall rotation mode was removed by subtracting ∑*θ*(*s*(*i*))/*N* from each angle. The shape covariance matrix *C*(*s*, *s*′) = 〈(*θ*(*s*)–〈*θ*〉)(*θ*(*s*′)–〈*θ*〉)〉 was constructed from 9 freely crawling worms sampled at 4 Hz, for a period of 30 minutes (a total of 60,000 images). Each eigenworm *u*_μ_(*s*) is an eigenvector of the covariance matrix ∑*_s_*_′_ C(*s*, *s*′)*u*_μ_(*s*′) = *λ*_μ_ *u*_μ_(*s*). The fractional variance captured by *K* eigenvectors is thus 
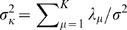
, where *σ* ^2^ = *Σ* *_μ_λ_μ_* is the total variance of the measurements. The same eigenworms shown in Figure 2 were used throughout the various analysis reported in the paper. The worm's phase was defined as *φ* = tan^−1^(−*a*_2_/*a*_1_) where *a*_1_ and *a*_2_ were both normalized to unit variance. The crawling speed was defined as the time derivative of the worm's center of mass.

### Equations of Motion

For the analysis of phase dynamics we sampled the worm shape at 32 Hz. Data for the construction of the equations of motion came from 12 worms, 5 trials per worm, with 4000 frames per trial. We also filtered each mode time series through a low-pass polynomial filter so that for each frame (26≤*m*≤3974), 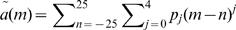 where {*p*_j_} are the best-fit polynomial coefficients. Mode time derivatives were calculated using derivatives of the polynomial filter. None of our results depend critically on the properties of the filter. The Langevin equations governing the phase dynamics are shown Eq. (4) and we learn the functions {*F*(*φ*,*ω*),*σ*(*φ*,*ω*)} directly from the time series [42],[43]. By construction 〈*σ*[*φ*(*t*),*ω*(*t*)]*η*(*t*)〉 = 0 and therefore the optimal rms estimate of *F*(φ,*ω*) is the conditional mean 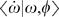. We estimate *F* by assuming a functional expansion 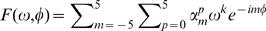, where the model parameters {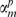} were determined by minimizing the rms error 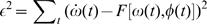 on training data (90%) and the hyperparameters {*m*_max_ = 5, *p*_max_ = 5} were chosen to minimize error on held-out data (10%). Once *F* is known we can determine the noise in the system; we normalize 〈*η* ^2^〉 so that 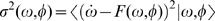. The attractors contained within our derived dynamics were obtained by evolving initial conditions spanning the sampled {*ω*, *φ*} plane for long times (93.75 s≈47 cycles). In the deterministic dynamics all trajectories evolve to one of four asymptotic states and we observed no switching.

### Thermal Impulse Response (Experiment)

Worms were prepared as described earlier but raised at a lower temperature (17°C) leading to a lower average ω before the thermal stimulus. A collimated beam with a 1/e diameter of 5.6 mm (standard stimulus) or 1.5 mm (painful) from a 1440 nm diode laser (FOL1404QQM, Fitel, Peachtree City, GA) was positioned to heat the area covering the worm. The diode laser was driven with a commercial power supply and controller (Thorlabs, Newton, NJ). Power and duration of the beam was controlled through software using LabVIEW. For each worm, 1000 seconds of data was collected in cycles of 50 seconds. 12.5 seconds into each cycle the laser was turned on for a duration of 75 ms at 150 mW (standard) or 250 ms at 100 mW (painful). The temperature increase caused by the laser pulses was measured using a 0.075mm T-type thermocouple (coco-003, Omega, Stamford, CT) placed on the surface of the agar and sampled with a thermocouple data acquisition device (USB-9211, National Instruments). For each measurement, 60 trials of 30 s cycles were averaged. The temperature increase was calculated by subtracting the maximum temperature (recorded immediately after the laser pulse) from the baseline temperature (recorded 9 s after the laser pulse). The temperature increase for the standard pulse was 0.12°C and the increase of the painful pulse was 0.73°C.

### Thermal Impulse Response (Analysis)

Data were taken from a collection of 13 worms, each stimulated with 20 repetitions of a Δ*T* = 0.1°C pulse. In Figure 6A, the time-dependent probability density *ρ*_t_(*ω*) was smoothed before the onset of the impulse with a gaussian low-pass filter of size 0.19 s in the *t* direction and 0.17 cycles/s in the *ω* direction. In Figure 6B the correlation function 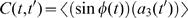 was calculated as follows. Far from the time of the impulse (frames 800 to 1574, impulse on frame 400), we expect time-translation invariance *C*_post_(*t*, *t*′) = *g*(*t*–*t*′)+ξ_post_(*t*, *t*′) where g(Δ) = 〈C(*i*,*j*)〉*_i_*_–*j = *Δ_ is the true correlation function and ξ_post_(*t*,*t*′) characterizes statistical error. Similarly in a time window around the impulse (frames 24 to 800), *C*_stim_(*t*, *t*′) = *g*(*t–t*′)+ξ_stim_(*t*, *t*′). However, the thermal impulse breaks this invariance and ξ_stim_(*t*, *t*′) contains both sampling fluctuations and stimulus-dependent correlation dynamics. To separate these effects we use singular value decomposition to compare ξ_post_ and ξ_stim_. We write each matrix ξ_post/stim_(*t*, *t*′) = ∑_t″_ *U*_post/stim_(*t*, *t*″)*S*_post/stim_(*t*″, *t*″)*V*_post/stim_(*t*″, *t*′) and find that only two singular values of ξ_stim_ are significantly larger than ξ_post_. We then reconstruct the two-point function around the stimulus as 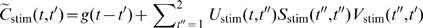.

### Thermal Steering

Preparation of worms and instrumentation were the same as described for the thermal impulse response. However, instead of processing worm images off-line, real-time calculation of the eigenworms and shape phase *φ* was done using custom dynamic-linked image processing libraries written in C along with supporting LabVIEW code. The modes were computed as previously described except that the spline interpolation algorithm was replaced with a Hermitian interpolation algorithm to reduce the processing time. The processing time was short enough to simultaneously track and calculate modes at 8 Hz. For phase dependent measurements, the laser was fired when the worm was moving forward and *φ* fell within a prescribed interval (width 1 radian). The laser pulse (150 mW) lasted for 75 ms and caused a temperature increase of 0.12°C. For each run a pair of triggering phase windows (0 to −1, and 2.1 to 3.1 radians) corresponding to the dorsal- and ventral-directed head swing was used. The sequence of each run started with a 5 minute period of no stimulus followed by the pair of phase dependent stimuli. The order of each pair of stimulus conditions was switched for each successive run. For the randomized pulse control experiments, the laser was fired with a uniform phase probability, but with conditions that restricted the firing interval to be longer than 2 seconds.

### Steering and Turn Identification

The time-average change in orientation of the worm's path, 〈
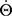
〉 (rad/s), was calculated from the angular changes between the positions of the center of mass of the worm during forward runs of at least 4 s in length. Given positions (*r*_1_, *r*_2_, *r*_3_,…, *r_N_*), the angles between connecting segments (*r*_2_– *r*_1_, *r*_3_– *r*_2_, *r*_4_– *r*_3_,…, *r_(N-1)_*) were calculated. 〈
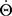
〉 was calculated in intervals of 10 s. Since the distributions were Gaussian (data not shown) with similar variance, we used the Student's t-test to determine if the values of 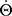 under thermal stimulation were significantly different than the control (*p*<0.05). Since we were interested in the change in orientation during forward motion we excluded trajectory data that contained large turns or reversals along with angular changes greater than π/4 radians. These events were automatically detected by measuring the compactness of the worm shape. Compactness was calculated by measuring the longest distance between two points in the worm shape (also known as the max feret distance) and normalizing this with the maximum value for the entire data run. Turns were flagged when the compactness fell below 0.6.
